# Timing matters: species-specific interactions between spawning time, substrate quality, and recruitment success in three salmonid species

**DOI:** 10.1002/ece3.1128

**Published:** 2014-06-10

**Authors:** Katharina Sternecker, Marco Denic, Juergen Geist

**Affiliations:** 1 Aquatic System Biology Unit, Department of Ecology and Ecosystem Management, Technische Universität MünchenD-85350, Freising, Germany; 2 Department of Anatomy, Ludwig-Maximilians-UniversitätD-80336, Munich, Germany

**Keywords:** Colmation, evolution, habitat quality, *Hucho hucho*, interstitial water, life-history strategy, reproduction, *Salmo trutta*, spawning season, stream substratum

## Abstract

Substratum quality and oxygen supply to the interstitial zone are crucial for the reproductive success of salmonid fishes. At present, degradation of spawning grounds due to fine sediment deposition and colmation are recognized as main factors for reproductive failure. In addition, changes in water temperatures due to climate change, damming, and cooling water inlets are predicted to reduce hatching success. We tested the hypothesis that the biological effects of habitat degradation depend strongly on the species-specific spawning seasons and life-history strategies (e.g., fall- vs. spring-spawners, migratory vs. resident species) and assessed temperature as an important species-specific factor for hatching success within river substratum. We studied the species-specific differences in their responses to such disturbances using egg-to-fry survival of Danube Salmon (*Hucho hucho*), resident brown trout (*Salmo trutta fario*), and migratory brown trout (*Salmo trutta lacustris*) as biological endpoint. The egg incubation and hatching success of the salmonids and their dependence on temperature and stream substratum quality were compared. Hatching rates of Danube salmon were lower than of brown trout, probably due to higher oxygen demands and increased interstitial respiration in spring. Increases in maximum water temperature reduced hatching rates of resident and migratory brown trout (both fall-spawners) but were positively correlated with hatching rates of Danube salmon (a spring-spawner). Significantly longer incubation periods of resident and migratory brown trout coincided with relatively low stream substratum quality at the end of the egg incubation. Danube salmon seem to avoid low oxygen concentrations in the hyporheic zone by faster egg development favored by higher water temperatures. Consequently, the prediction of effects of temperature changes and altered stream substratum properties on gravel-spawning fishes and biological communities should consider the observed species-specific variances in life-history strategies to increase conservation success.

## Introduction

Salmonid fishes are adapted to cool, oligotrophic rivers, and lakes. Throughout their distribution range, they are considered target species in conservation with a high ecologic and socio-economic value. For instance, species such as the Danube salmon (*Hucho hucho* L.) are top predators of their ecosystems with important regulatory functions (Geist et al. 2009). In general, salmonids are main target species for aquaculture and recreational fishing (Denic and Geist 2010). Some species like brown trout (*Salmo trutta* L.) or Atlantic salmon (*Salmo salar* L.) are obligate hosts for the larvae of the freshwater pearl mussel (*Margaritifera margaritifera* L.) (Young and Williams 1984; Geist et al. 2006; Taeubert et al. 2010). Consequently, there is a high interest in stable and healthy salmonid populations, resulting in a diversity of habitat assessment and restoration efforts globally (Denic and Geist 2010; Pander and Geist 2013; Sternecker et al. 2013a). Habitat restoration is required, because most salmonid species have severely declined in recent decades and are currently endangered (e.g., Kondolf 1997; Jungwirth et al. 2003; Thorstad et al. 2008; Geist 2011; Kemp et al. 2011).

Salmonids are lithophilic fish species, that is, they deposit their eggs in the interstitial zone of suitable gravel banks. For successful reproduction, all salmonid species depend on clean stream substratum (i.e., with low fine sediment content) with an intact and well-oxygenated interstitial zone for egg and larval development (Crisp 1996; Rubin and Glimsäter 1996; Kondolf 2000; Ingendahl 2001; Malcolm et al. 2003; Greig et al. 2007; Sternecker and Geist 2010; Sternecker et al. 2013a,b).

Several studies recognized fine sediment introduction and colmation (i.e., blockage of streambed interstitial spaces by the ingress of fine sediments and organic material; Buss et al. 2009) of spawning gravels as major factors for reproductive failure (Jungwirth 1978; Acornley and Sear 1999; Soulsby et al. 2001; Levasseur et al. 2006; Franssen et al. 2012). Due to the influence of temperature on oxygen solubility in water and on salmonid egg development, rising water temperatures in the course of global warming, damming, and cooling water inlets are expected to further reduce salmonid reproduction rates (Lake et al. 2000; Battin et al. 2007; Jonsson and Jonsson 2009).

However, different species and evolutionary significant units (Moritz 1994) of salmonids use different time periods for spawning and may thus be differently affected by changes in their spawning habitats. Such differences among populations of Pacific Chinook salmon (*Oncorhynchus tshawytscha*) have already been observed in previous studies (e.g., Crozier and Zabel 2006), but remain untested for Atlantic salmonids. Such species-specific differences of life-history strategies are well known (e.g., Sternecker and Geist 2010), but the potentially resulting differences in the population-level responses in spring- versus fall-spawning salmonids have not been considered in the context of habitat degradation. In Germany, the Danube Salmon (*Hucho hucho*) is a migratory spring-spawner, whereas the resident brown trout (*Salmo trutta fario*) and the migratory brown trout (*Salmo trutta lacustris*) are typical fall-spawners. As a consequence of their individual life-history strategies, the reproductive periods of the study species do not overlap in time. Thus, all three salmonid species face different environmental conditions during reproduction.

In the current study, we investigated to what extent different physical impacts on reproductive success are species-dependent by comparing data of monitoring experiments in three native European salmonids: the Danube Salmon, resident brown trout and migratory brown trout. We tested the hypotheses that (1) Danube salmon is less affected by increased fine sediment deposition and colmation than migratory and resident brown trout due to shorter egg development periods and (2) increasing water temperatures reduce hatching rates of the study species.

## Materials and Methods

### Study area

**Figure 1:**
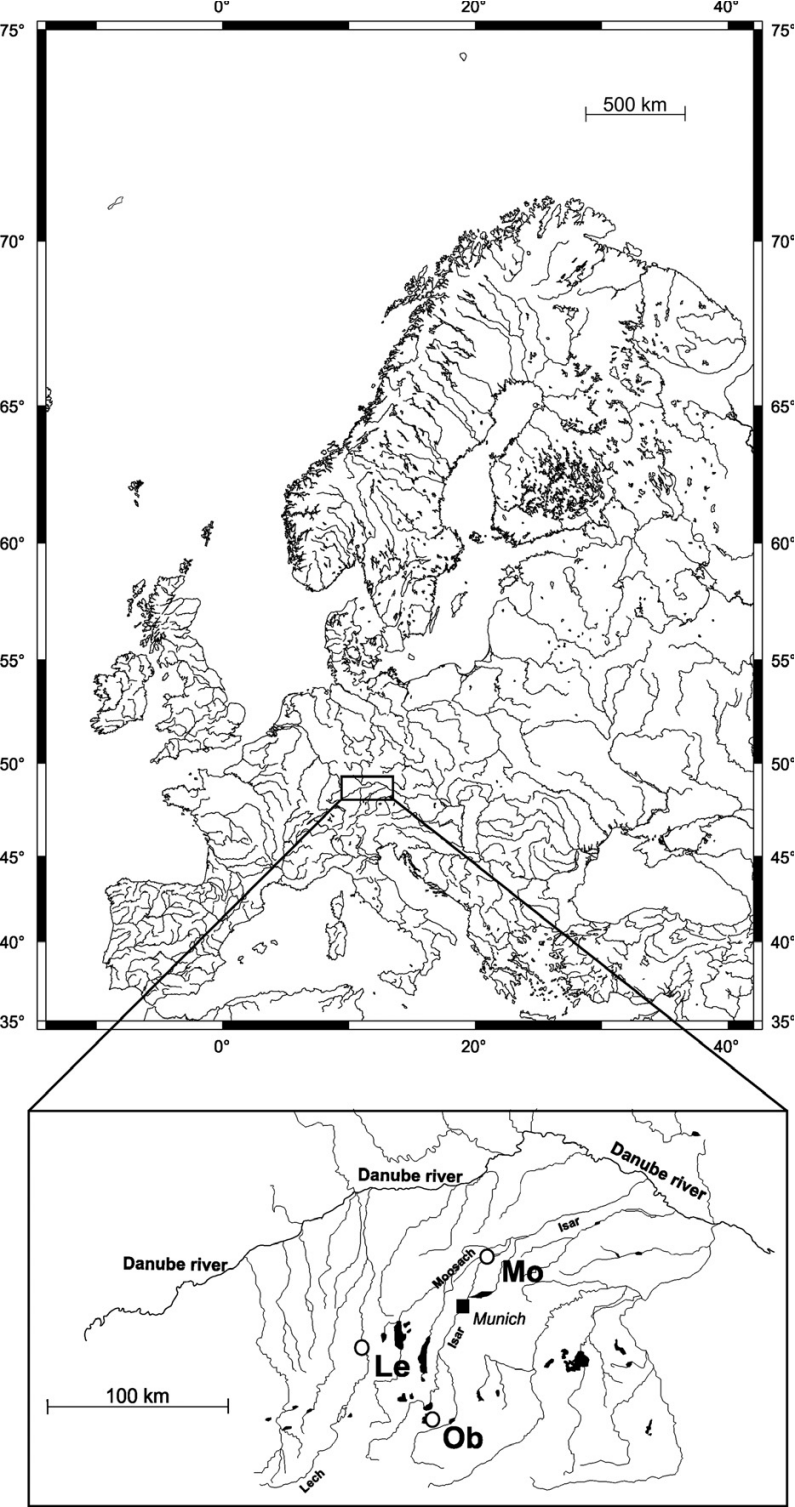
Location of the study sites in the rivers Lech (Le), Moosach (Mo) and Obernach (Ob).

Three typical alpine salmonid streams in Bavaria, Germany, inhabited by all three study species, were selected for this case study (Fig. 1). The study streams were the rivers Lech, Moosach and Obernach (mean annual discharges: 82.9 at water gauge Landsberg, 2.6 and 1 m^3^·s^−1^, respectively). They are all anthropogenically manipulated limestone streams within the Danube catchment, with regulated flow regimes and migration barriers due to dams and hydropower generation. In all three rivers, a decline of salmonid recruitment during the last decades was observed.

Spawning grounds, where natural reproduction of at least one of the study species was observed, were chosen as study sites and could be natural or man-made gravel banks (Pulg et al. 2013; Sternecker et al. 2013b).

### Microhabitat assessment

Differences in spawning habitat quality were analyzed using egg-to-fry development success (active bioindication) as a biological endpoint (for details, see Sternecker et al. 2013b). A total of 77 egg sandwich boxes (ES; Pander et al. 2009) were exposed during three consecutive years and spawning seasons (2009–2011). Of these ES, 37 were filled with Danube salmon eggs (16 × River Lech, 21 × River Moosach), 29 with eggs of resident brown trout (10 × River Lech, 19 × River Moosach), and 12 with eggs of migratory brown trout (River Obernach). Each ES was filled with 90 salmonid eggs in separate chambers and buried in the substratum to line up with the substratum surface, that is, 30 eggs were exposed in substratum depths 0–50 mm, 50–100 mm, and 100–150 mm, respectively. The ESs were placed haphazardly in the gravel banks in the first studied spawning season. During the consecutive spawning seasons, the location of the ESs was chosen on the basis of the first season. For winter bioindication, 5–12 female and 3–6 male resident brown trout (RBT) of a hatchery stock (Landesfischzuchtanstalt Mauka, Massenhausen, Germany) and two females and three males of autochthonous migratory brown trout (MBT) from Lake Walchensee (River Obernach is a tributary of Lake Walchensee) were used as spawners. For spring bioindication, two female and three male Danube Salmon (DS) of the hatchery stock of the “Fischereilicher Lehr-und Beispielbetrieb Lindbergmühle” were used as spawners. In each experiment, fertilized eggs of different spawners were mixed and randomly distributed to ES. ES were exposed considering local stream bed variability and the avoidance of spatially autocorrelated datapoints (Braun et al. 2012). Hatching (egg-to-fry development) success of substratum exposures was analyzed after hatching in field references (anchored floating box with 3 × 100 eggs each study site) was observed (according to Sternecker et al. 2013b). Field references allowed to observe egg development without disturbing ES experiments and to detect hatching success under river water conditions without the effects of stream substratum during the egg incubation.

Interstitial water samples for water quality analysis were taken from the measurement unit of each ES at 50 mm, 100 mm, and 150 mm substratum depth. Interstitial water conditions were characterized by analyzing dissolved oxygen concentration (mg·L^−1^), pH, specific conductance (corrected to 20°C), and redox potential (mV) within water samples using handheld oxygen-, conductivity-, and pH-meters (WTW, Weilheim, Germany). Nitrate 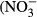, mg·L^−1^), nitrite (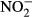, mg·L^−1^), and ammonium (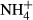, mg·L^−1^) were determined by using analytical kits (Spectroquant, Merck, Germany) and a PC spectrometer (photoLab S12, WTW). Next to every ES, redox potential was measured in three substratum depths (50 mm, 100 mm, and 150 mm) according to Geist and Auerswald (2007). Water temperature during egg incubation was continuously (one data point per hour) monitored by data loggers (EL-USB-1, Lascar Electronics, Salisbury, UK). All other parameters were measured three times, that is, at the beginning of egg exposure, after eggs reaching the eyed stage and after hatching of juveniles. Habitat quality in the hyporheic zone is determined by the exchange rate with the free-flowing water, which is reduced by fine sediments clogging interstitial macropores. This causes deviances in physicochemical parameters between the hyporheic zone and the free-flowing water. Differences of exchange rates were evaluated by additional measurements of all physicochemical parameters in the free-flowing water proximal to each ES (Sternecker et al. 2013b).

### Statistical analysis

Differences in hatching success (egg-to-fry survival) between species, as well as between rivers, were analyzed using time and water temperature as determining variables. Duration of the incubation period was expressed in absolute values of days (d) and using sums of degree days (dd). Dd were calculated as the product of mean water temperature during egg incubation and absolute incubation period in days. Bivariate correlations were calculated between hatching rate, mean and maximum water temperature during the incubation period, respectively. Relative hatching rate (calculated for each ES as a proportional hatching rate of eggs) was used for the following calculations as proposed in the study of Sternecker et al. (2013b). Delta values were calculated by the difference between the value in the free-flowing water and in the interstitial water, respectively. For analyzing the differences of interstitial water conditions between the rivers Moosach, Obernach and Lech during different spawning seasons, discriminant analyses (DCA) were conducted to separate groups of high- and low-hatching success for the individual rivers and individual salmonid species, respectively.

For the evaluation of the interstitial flow-through, the angles between discriminant functions (considering absolute and delta values separately) were calculated according to Batschelet (1979). High- and low-hatching success of every individual species was determined for DCA using cluster analyses as the distribution of hatching success varied between species. Differences between species, spawning season, study river, and study year were calculated using Mann–Whitney *U*-tests. All statistical analyses were performed using the software IBM SPSS Statistics 20 (IBM Corporation, Armonk, NY).

## Results

**Figure 2:**
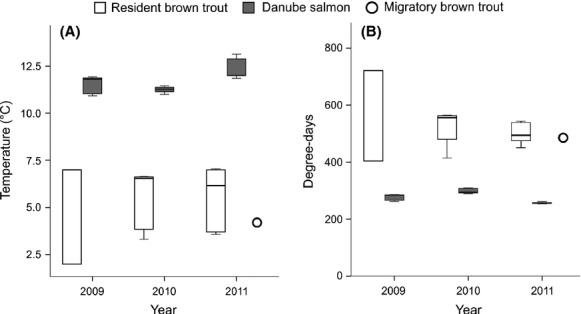
(A) Mean temperature [°C] during salmonid egg development, and (B) period of egg development (sum of degree-days) in three spawning seasons for Danube salmon (*Hucho hucho*) in the rivers Lech (*n*_2009_ = 6, *n*_2010_ = 7 and *n*_2011_ = 6) and Moosach (*n*_2009_ = 7, *n*_2010_ = 8 and *n*_2011_ = 8), for resident brown trout (*Salmo trutta* fario) in the rivers Lech (*n*_2009_ = 6, *n*_2010_ = 4 and *n*_2011_ = 3) and Moosach (*n*_2009_ = 8, *n*_2010_ = 7 and *n*_2011_ = 6) and for migratory brown trout (*Salmo trutta lacustris*) in river Obernach (*n*_2011_ = 1), respectively (Box-Whisker plots; Whiskers: maximum, minimum; Box: 0.25 quartile, median and 0.75 quartile).

Egg incubation period, degree days until hatch, and mean water temperature during egg incubation varied with species, spawning season, study river, and study year (Fig. 2). The differences between spring-spawning DS and fall-spawning MBT as well as RBT were highly significant (Mann–Whitney *U*-tests, *P* < 0.001 for each parameter, respectively), whereas the conditions of egg development of RBT and MBT were comparable with similar egg incubation periods, degree days until hatch and water temperatures (Table 1).

**Table 1:** Temperature, incubation period, and hatching success of salmonids species. Temperature (mean, minimum and maximum), sum of degree-days as well as incubation period (days) and hatching rate of resident brown trout (*Salmo trutta fario*), Danube salmon (*Hucho hucho*) and migratory brown trout (*Salmo trutta lacustris*); means are provided with standard deviations; the number of data loggers for measuring temperature was *n* = 15 (river Moosach) and *n* = 8 (river Lech) for resident brown trout, *n* = 18 (river Moosach) and *n* = 22 (river Lech) for Danube salmon and *n* = 1 (river Obernach) for migratory brown trout

River	Species	Mean temperature (°C)	Minimum temperature (°C)	Maximum temperature (°C)	Mean sum of degree-days (°C)	Mean incubation period	Hatching rate (%)
Moosach	*Salmo trutta fario*	7 ± 0.3	3	15	554 ± 55.3	84 ± 5.8	31 ± 34.9
Lech	*Salmo trutta fario*	3 ± 0.6	1	9	453 ± 36.5	126 ± 32.5	56 ± 22.6
Moosach	*Hucho hucho*	12 ± 0.3	8	17	277 ± 17.2	24 ± 2.0	37 ± 27.1
Lech	*Hucho hucho*	12 ± 0.8	9	16	280 ± 23.1	24 ± 2.0	18 ± 19.3
Obernach	*Salmo trutta lacustris*	4	2	8	485	116	75 ± 25.8

Brown trout hatching success in field references was 38–89% in the river Moosach (RBT), 71–98% in the river Lech (RBT) and 96% in the river Obernach (MBT). The DS hatching success in field references exposed to open water, that is, excluding substrate effects investigated with the ES exposures, was 70–83% in the river Moosach and 41–84% in the river Lech. Overall, ES exposure hatching rates of MBT (75%) were significantly higher than the RBT hatching rates (42%) and DS hatching rates (30%). When ES exposure hatching rates were analyzed river specifically, RBT hatching rates in the River Lech were similar to MBT hatching rates. DS hatching rates in the River Lech were significantly lower than in all other groups, except for RBT in the River Moosach. Regarding the studied species, the impact of stream substratum quality acted longer during the fall spawning season than in spring, as evident from the extended egg incubation periods of RBT and MBT compared with DS. Mann–Whitney *U*-tests revealed significant differences for RBT (*P* < 0.001), but not for DS (*P* > 0.569) concerning egg incubation periods, sums of degree days until hatching and mean water temperatures between the rivers Moosach and Lech. These patterns remained highly similar between study years.

High rates of correct classification of hatching rates by DCA into high and low ES exposure hatching success groups indicate that a linear combination of interstitial water parameters successfully separates the two groups (Table 2). In the discriminant function, positive coefficients with high values were evident for oxygen concentration and redox potential, stressing the importance of these two variables. The weight and direction of action (positive or negative effect) of other coefficients varied by species. For instance, coefficients of MBT and RBT were similar to each other, whereas coefficients of specific conductance varied among DS compared with MBT and RBT (Table 3). The correlation between discriminant functions of absolute and delta values (differences between interstitial and free-flowing water) was mostly low. The angle calculated between discriminant functions of absolute and delta values was highly variable with respect to species. An angle of *θ* = 78.3° indicated that differences between free-flowing and interstitial water were most substantial for RBT. The angles for functions of MBT and DS were smaller with *θ* = 53.1° and *θ* = 46.2°, respectively. Yet, similarity of conditions of free-flowing and interstitial water differed substantially on the river scale, for example, the angle of DS discriminant functions were *θ* = 66.7° in the River Moosach and *θ* = 48.7° in the River Lech.

**Table 2:** Classification of the discrimination analysis (DCA); DCAs refer to the dependency of physicochemical parameters [O_2_ = dissolved oxygen concentration (mg·L^−1^), pH, specific conductance (*μ*S·cm^−1^; corrected to 20°C), Eh = redox potential (mV), 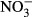 = nitrate (mg·L^−1^), 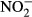 = nitrite (mg·L^−1^) and 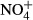 = ammonium (mg·L^−1^)] on hatching success (relative rate) in the river Moosach (2009–2011), the river Lech (2009–2011), in both rivers (macro-scaled level) and the river Obernach (2011); absolute values and delta values of the physicochemical parameters were considered separately; delta values of the physicochemical parameters were calculated by the difference between interstitial and free-flowing water

			Abs. values		Delta values	
**RBT**		Predicted class				
Moosach and Lech			HHR	LHR	HHR	LHR
	Actual class	HHR	95.1	4.9	89.4	10.6
		LHR	21.2	78.8	41.7	58.3
		% explained variance	87.8		75.9	
Moosach		Predicted class				
			HHR	LHR	HHR	LHR
	Actual class	HHR	92.0	8.0	88.0	12.0
		LHR	22.6	77.4	29.0	71.0
		% explained variance	83.9		78.6	
Lech		Predicted class				
			HHR	LHR	HHR	LHR
	Actual class	HHR	93.8	6.2	90.9	9.1
		LHR	0.0	100.0	0.0	100.0
		% explained variance	94.4		92.6	
**DS**		Predicted class				
Moosach and Lech			HHR	LHR	HHR	LHR
	Actual class	HHR	56.1	43.9	38.6	61.4
		LHR	32.1	67.9	11.3	88.7
		% explained variance	61.8		62.7	
Moosach		Predicted class				
			HHR	LHR	HHR	LHR
	Actual class	HHR	80.8	19.2	69.2	30.8
		LHR	29.7	70.3	40.5	59.5
		% explained variance	74.6		63.5	
Lech		Predicted class				
			HHR	LHR	HHR	LHR
	Actual class	HHR	61.3	38.7	74.2	25.8
		LHR	6.2	93.8	12.5	87.5
		% explained variance	72.3		78.7	
**MBT**		Predicted class				
Obernach			HHR	LHR	HHR	LHR
	Actual class	HHR	90.0	10.0	75.0	25.0
		LHR	0.0	100.0	0.0	100.0
		% explained variance	91.2		77.8	

HHR, high hatching rate; LHR, low hatching rate.

**Table 3:** Effects of physicochemical parameters on hatching success of different salmonid species. Discriminant analysis (DCA) referring to the dependency of physicochemical parameters on hatching success (relative hatching rate) of RBT, DS and MBT in the rivers Moosach (2009–2011), Lech (2009–2011) and Obernach (2011), respectively. Groups were defined by cluster analysis (high- vs. low-hatching success), discriminatory power of absolute values as well as delta values of the physicochemical parameters (difference between interstitial and free-flowing water) were compared

	RBT		DS		MBT	
			
Variables	Abs. values	Delta values	Abs. values	Delta values	Abs. values	Delta values
	Moosach and Lech				Obernach	
Redox potential	0.722	0.779	0.586	0.729	0.457	0.279
Oxygen concentration	0.427	0.223	0.352	0.770	0.374	0.523
Specific conductance	−0.435	0.422	0.640	0.267	0.183	−0.386
pH	0.338	0.362	−0.144	0.701	0.397	0.151
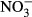 concentration	−0.440	0.292	0.606	0.307	0.275	0.089
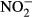 concentration	−0.332	0.637	0.142	0.074	−0.114	−0.143
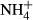 concentration	0.045	0.097	−0.529	0.408	−0.089	0.172
	Moosach					
Redox potential	0.596	0.755	−0.387	0.614		
Oxygen concentration	0.783	0.532	−0.494	0.375		
Specific conductance	0.386	−0.025	0.472	0.268		
pH	0.407	0.245	−0.222	−0.056		
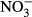 concentration	0.250	−0.130	0.277	0.385		
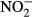 concentration	0.439	−0.301	0.393	0.284		
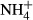 concentration	−0.051	0.195	0.602	0.103		
	Lech					
Redox potential	0.287	0.438	0.354	0.376		
Oxygen concentration	0.091	−0.079	0.868	0.667		
Specific Conductance	0.235	−0.397	−0.304	−0.245		
pH	0.394	−0.105	0.737	0.613		
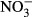 concentration	0.030	−0.115	0.119	0.660		
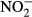 concentration	−0.304	0.077	−0.070	0.075		
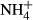 concentration	−0.387	0.473	−0.280	0.257		

The correlation between mean water temperature and hatching rate was weak, but the correlation between maximum water temperature during egg incubation and hatching rate was significant. There was a strong negative correlation for MBT and RBT (Spearman/Rho −0.711; *P* < 0.001) and a positive one for DS (Spearman-Rho 0.529; *P* = 0.001).

## Discussion

The results of this study suggest that the differences in life-history strategies of salmonid fish species result in different susceptibilities to substrate degradation and temperature change. Habitat degradation is thus likely to exert diverse mechanisms and directions of selection on different salmonid species. There is evidence that substratum degradation lowered reproductive success in all study species. In contrast, temperature increases reduce hatching rates of fall-spawning MBT and RBT but are positively correlated with spring-spawning DS-hatching rates. Consequently, the precise analysis of spawning habitat deficits (e.g., lack of stream substratum, substratum colmation or migration barriers) with respect to species-specific variances in life-history strategies is crucial for the success of conservation management.

### Differences in evolutionary effects of stream substratum and temperature on salmonid recruitment

The period of egg and larval development within the substrate is crucial for reproductive success. Redegradation processes (e.g., fine sediment introduction) after redd building induce compaction of substratum, which affects interstitial water conditions, especially toward the final stages of the egg and larval development (Zeh and Doenni 1993; Peterson and Quinn 1996a; Rubin et al. 2004; Julien and Bergeron 2006; Jensen et al. 2009; Sternecker et al. 2013b). We showed that the egg development of DS is significantly faster than of RBT and MBT. Consequently, the period of time in which DS hatching success is affected by stream substratum degradation is shorter than of MBT and RBT. A shorter period of development in substratum by DS compared with RBT in the subsequent life stage, that is, the emergence of fry, was shown previously (Sternecker and Geist 2010). Cumulative effects of shorter development times are important to consider, because it has also been shown that an earlier emergence of fry increases competitiveness of species and individual fish, respectively (Skoglund et al. 2011). As both life stages (egg-to-fry and emergence of fry) benefit from an accelerated development, the total effect is likely to be even stronger than the one described herein. The hypothesis that DS is adapted to high-quality substratum was confirmed by the smaller differences between interstitial and free-flowing water conditions compared with MBT and RBT at the end of egg development. The hatching success of spring-spawning DS was lower than the hatching success of fall-spawning brown trout at the same gravel banks. The reduced hatching rates of DS in the hyporheic zone suggest that DS have even higher spawning habitat quality requirements than RBT or MBT. Spring-spawners seem to be more strongly affected by substratum colmation which is also supported by the more serious decline of spring runs compared with fall runs in California Chinook salmon populations (Fisher 1994).

Although high DCA coefficients for oxygen content and redox potentials indicated that oxygen availability is important for all studied species, coefficients were highest for DS suggesting either extremely high oxygen demand of DS or increased oxygen depletion due to higher water temperatures and biological activity in spring. However, a high number of eggs and big redd sizes caused by the large body size of the female DS likely increase the probability of eggs within high substratum quality pockets on the microscale, because the eggs are distributed within a bigger area in the substratum. A high microscale variability of interstitial water conditions during egg development was previously detected (Malcolm et al. 2009; Sternecker et al. 2013b) and seems to be characteristic of the hyporheic zone of most stream ecosystems (Braun et al. 2012). Such variation at small spatial scales was recently described to buffer temporal fluctuations in early juvenile survival in Pacific Chinook salmon (Thorson et al. 2014). Substratum depth in our study was limited to 150 mm, hence deeper zones with a potential flow-through of oxygen rich ground water was not considered resulting in a possible underestimation of hatching success (Peterson and Quinn 1996b). Furthermore, individual adaptation can moderate the effects of increasing fine sediment input or other consequences of climate change, as for example the burial depth of salmonid eggs is known to vary between individuals and populations within salmonid species (DeVries 1997). Hendry and Day (2003) hypothesized that a large egg size is advantageous during low oxygen supply within stream substratum. As a consequence, smaller fish with smaller eggs should be subject to stronger selective pressure than larger fish. Consequently, larger size of the DS females compared with brown trout females may be an evolutionary result of more adverse interstitial water conditions in spring.

The results of this study indicate that RBT and MBT (both fall-spawners) depend more strongly on cool water temperatures, which was suggested by the strong negative correlation between maximum water temperatures and hatching success in our study. This makes them more susceptible to the effects of climate change, which is expected to cause more frequent temperature peaks in winter time in the future (Mauser et al. 2008). Additionally, these winter temperature peaks result in untimely snow melts and increased fine sediment mobilization after rain events due to low vegetation cover in catchments (Herringshaw et al. 2011). As a consequence, the risks of elevated fine sediment input into spawning grounds and of river bed scouring increase, which both negatively affect hatching success of fall spawners (Battin et al. 2007; Jonsson and Jonsson 2009; Wedekind and Küng 2010; Goode et al. 2013).

Altogether, the salmonid species in this study seem to be equally affected by temperature changes and reduced water quality in the interstitial zone. However, DS is currently more endangered than brown trout, most likely due to anthropogenic impacts that additionally affect this species (IUCN 2013). In particular, DS spawning migrations are hampered by habitat fragmentation, for example, due to dams and hydroelectric power stations. A separate consideration of brown trout evolutionary significant units RBT and MBT further supports this hypothesis. MBT, which is a migratory form of brown trout, is endangered throughout most of its distribution range, whereas RBT lives stationary and shows the most stable populations of the studied species/evolutionary significant units (Lelek 1987; Kottelat and Freyhof 2007; Denic and Geist 2010).

### Management implications

Selective forces on egg burying fish species and habitat degradation factors often have synergistic effects (Parrish et al. 1998; Lake et al. 2000). Such an adverse synergistic effect of stream substrate degradation and increased water temperature on salmonids was detected in our study and found to be species-specific. It is likely to be driven by variable and scale-dependent physicochemical and genetic impacts (Sternecker et al. 2013b; Thorson et al. 2014). Consequently, there is no general solution for the support of declining salmonid populations, as restoration concepts have to consider specific conditions of the river (stretch) to be restored as well as specific demands of the target species.

The examples in this study reveal that reasons for the lack of suitable spawning substrates can vary, which requires the use of different restoration techniques. In rivers like the Moosach, where substrates are clogged with fine material and often colmated, cleaning, and loosening of substrates is an efficient restoration method (Shackle et al. 1999; Pulg et al. 2013; Sternecker et al. 2013a). On the other hand, in rivers with interrupted bedload transport such as the river Lech, the addition of clean gravel is recommended (Pulg et al. 2013). It has to be noted that in the course of spawning ground restoration, species-specific habitat preferences have to be considered as suitable gravel size, flow velocity and water depth at spawning grounds are fish size and species dependent (Crisp and Carling 1989; Kondolf and Walman 1993; Louhi et al. 2008). Thus, systematic comparisons of different types of substrate restoration are mandatory for the assessment of their sustainability (Mueller et al. 2014). Substratum in the river Obernach proved to be of high quality, hosting a healthy RBT population but allowing no recruitment of MBT (Denic and Geist 2010), which underlines the importance of habitat connectivity for migratory species. In case of migratory species like MBT and DS, our study clearly shows that river continuity is to be set as a first priority, to make suitable spawning habitats accessible. The migration to the spawning habitat is often interrupted by barriers that impair the return of mature adults. Where possible, barrier removal should be preferred, otherwise, the construction of fish ways is recommended (Ovidio and Philippart 2002; Gosset et al. 2006; De Leaniz 2008).

The widespread practice of breeding all of the studied fish species in aquaculture facilities and their consecutive stocking is likely to reduce fitness of individuals with possible negative effects on the natural recruitment of those species. The study of Geist et al. (2009) showed a high variance in the success of DS stocking measures indicated by a strongly variable contribution of hatchery stocks to the identified genetic clusters. Milot et al. (2013) demonstrated a reduced reproductive success of hatchery born Atlantic salmon (*Salmo salar*) compared with wild individuals. Furthermore, the later fish were released to natural habitats, the lower was their contribution to natural reproduction and a selection for other biological characteristics than such that are crucial for natural reproduction, for example, the egg and larval development within stream substratum was suggested. Genetic fitness of spawners may also have influenced the results of this study, as spawners from a wild stock (MBT) and from hatchery stocks were used (DS and RBT). However, the significant difference of DS hatching rates in the Rivers Moosach and Lech and the similarity between MBT hatching rates in the River Obernach and RBT hatching rates in the River Lech corroborate a dominant influence of habitat conditions on hatching success.
